# Different Active Microbial Communities in Two Contrasted Subantarctic Fjords

**DOI:** 10.3389/fmicb.2021.620220

**Published:** 2021-06-24

**Authors:** Claudia Maturana-Martínez, Camila Fernández, Humberto E. González, Pierre E. Galand

**Affiliations:** ^1^Sorbonne Université, CNRS, Laboratoire d’Ecogéochimie des Environnements Benthiques, Banyuls-sur-Mer, France; ^2^Centro de Investigación en Dinámica de Ecosistemas Marinos de Altas Latitudes and Universidad Austral de Chile, Valdivia, Chile; ^3^Sorbonne Université, CNRS, Laboratoire d’Océanographie Microbienne, Banyuls-sur-Mer, France; ^4^Departamento de Oceanografía and Centro de Investigación Oceanográfica COPAS Sur-Austral, Universidad de Concepción, Concepción, Chile

**Keywords:** RNA, DNA, glacier, fjord, Chile, subantarctic, bacteria, archaea

## Abstract

Microorganisms play a crucial role in biogeochemical processes affecting the primary production and biogeochemical cycles of the ocean. In subpolar areas, the increment of the water temperature induced by climate change could lead to changes in the structure and activity of planktonic microbial communities. To understand how the structure of the microbial community in Chilean Patagonian fjords could be affected by climate change, we analyzed the composition of the prokaryotic community (bacteria-archaea) in two fjords (Pia and Yendegaia) with contrasting morphological and hydrological features. We targeted both the standing stock (16S rRNA genes) and the active fraction (16S rRNA transcripts) of the microbial communities during two consecutive austral winters. Our results showed that in both fjords, the active community had higher diversity and stronger biogeographic patterns when compared to the standing stock. Members of the *Alpha*-, *Gamma*-, and *Deltaproteobacteria* followed by archaea from the Marine Group I (*Thaumarchaeota*) dominated the active communities in both fjords. However, in Pia fjord, which has a marine-terminating glacier, the composition of the microbial community was directly influenced by the freshwater discharges from the adjacent glacier, and indirectly by a possible upwelling phenomenon that could bring deep sea bacteria such as SAR202 to the surface layer. In turn, in the Yendegaia, which has a land-terminating glacier, microbial communities were more similar to the ones described in oceanic waters. Furthermore, in Yendegaia fjord, inter-annual differences in the taxonomic composition and diversity of the microbial community were observed. In conclusion, Yendegaia fjord, without glacier calving, represents a fjord type that will likely be more common under future climate scenarios. Our results showing distinct Yendegaia communities, with for example more potential nitrogen-fixing microorganisms (*Planctomycetes*), indicate that as a result of climate change, changing planktonic communities could potentially impact biogeochemical processes and nutrient sources in subantarctic fjords.

## Introduction

The Chilean Patagonia comprises one of the main fjord areas in the world (Iriarte et al., 2010). The numerous fjords are important ecosystems stressed by climate change, where freshwater input, ocean acidification, and water temperature directly impact the water column of these fragile environments (Torres et al., 2011; Iriarte et al., 2018, 2019). The functioning, hydrological structure, productivity levels, and dominant species of these marine and coastal systems are changing together with climate (Iriarte et al., 2010; González et al., 2013).

Two broad types of Patagonian fjords can be distinguished. Fjords of the temperate type, in which the water column does not freeze, and fjords of the subpolar type, in which water freezes in winter, but with average summer temperatures exceeding 0°C (Domack and Mcclennen, 1996; Gilbert, 2000; Howe et al., 2010). Both types of fjords may (or not) be associated with a glacier. Some of the associated glaciers can be land-terminating glaciers that are retreating and forming proglacial rivers that bring freshwater and terrestrial sediments to the fjords (Chu, 2013; Giesecke et al., 2019). Other glaciers are marine-terminating glacier where freshwater discharges occur directly from the glacier to the fjord surface, but also below (from 10 to 100 m), where the released meltwater can produce upwelling of deep sediments and nutrients (Chu, 2013; Giesecke et al., 2019). These two different types of freshwater inputs could have a distinct effects on the fjords’ ecology, including their productivity (Meire et al., 2017) and phytoplankton size-structure (Cuevas et al., 2019), which in turn could lead to modifications of the fjord trophic state (Montero et al., 2011).

Two marked layers characterize the water column of Patagonian fjords. One layer is superficial with colder and less saline water that usually has high concentrations of silicic acid and suspended sediment (Valdenegro and Silva, 2003; Sievers and Silva, 2008; Silva, 2008; González et al., 2010; Iriarte et al., 2014). The other layer is more profound and influenced by a constant influx of subantarctic oceanic waters (SAAW), which has higher salinity and nutrient concentrations (nitrate and phosphate) (Valdenegro and Silva, 2003; Sievers and Silva, 2008; Silva, 2008; González et al., 2013). In some cases, the exchange of oceanic and estuarine waters (EWs) can be limited by the presence of a sill at the mouth of fjords (Howe et al., 2010).

The distinct water masses characterizing fjords should promote the presence of distinct communities of planktonic microorganisms. Differences in distribution at the genus level have indeed been observed between the surface and bottom fjord waters in Svalbard, Norway (Zeng et al., 2009; Teske et al., 2011). Changes in the physical and chemical properties of the water column, as a consequence of freshwater discharge, can also lead to variations in the microbial community composition (Piquet et al., 2010, 2011; Zeng et al., 2013; Gutiérrez et al., 2015). Furthermore, high sediment concentrations induced by mixing water can limit light penetration into the water column, which in turn reduces the euphotic zone, leading to changes in the microbial community composition as shown in arctic fjords of Svalbard (Piquet et al., 2010). Studies on particulate matter and sediments in fjords have also evidenced that glacial discharge has the potential to transform the microbial community structure through the transport of particles as seen in the Arctic (Bourgeois et al., 2016; Jain et al., 2019). Variations in microbial diversity can affect the microbial activity and thereafter have an impact on biogeochemical cycles (Gutiérrez et al., 2018; Balmonte et al., 2019).

The impact of fjord hydrography on the microbial community structure is now better understood, but it remains to be studied thoroughly in the southern hemisphere. Besides, little is known about possible annual differences in community composition or differences between fjords from the same region. One study from Svalbard showed distinct communities between adjacent fjords (Piquet et al., 2010), but there is no information from Patagonian fjords as the number of researches on the marine prokaryotic community is limited (Vargas et al., 2008; Mackenzie et al., 2012; Ugalde et al., 2013; Gutiérrez et al., 2015, 2018; Undabarrena et al., 2016) in comparison to the northern and Arctic fjords (Zeng et al., 2009; Piquet et al., 2010, 2011; Zaikova et al., 2010; Teske et al., 2011; Piontek et al., 2013; Roy et al., 2013; Sperling et al., 2013; Winter et al., 2013; Cardman et al., 2014; Bourgeois et al., 2016; Sinha et al., 2017a,b; Balmonte et al., 2019). Bacterial communities from fjords in high latitudes are usually dominated by *Alphaproteobacteria*, *Gammaproteobacteria*, and *Bacteroidetes* (Zeng et al., 2009; Piquet et al., 2010; Gutiérrez et al., 2015). Among archaea, *Thaumarchaeota* (Marine Group I, MGI) appears as the most common in deep (Galand et al., 2008, 2009, 2010) and surface high latitude waters (Gutiérrez et al., 2018). However, the majority of the studies on prokaryotic community structure in high latitudes fjords have been performed on the total community (DNA fraction) (Zeng et al., 2009, 2013; Piquet et al., 2010; Teske et al., 2011; Gutiérrez et al., 2015, 2018) and little is known about how the active community (RNA fraction) respond to possible hydrographic variations.

The general aim of this study was to test if two Patagonian fjords with different geomorphological features had different microbial communities. We studied two fjords located along the Beagle Channel in the southern Chilean Patagonia: Pia fjord featuring a marine-terminating glacier, and Yendegaia fjord featuring a land-terminating glacier, which represents a fjord under future climate scenarios. More specifically we tested (i) if fjords with similar climate and localization, but with distinct freshwater inputs, have different prokaryotic communities, and (ii) if the prokaryotic community of Yendegaia fjord present inter-annual changes (July 2017 and July 2018). We analyzed both the total and active fraction of the communities, based on DNA and RNA, in relation to the physical, chemical, and biological properties of the water collected along horizontal and vertical transects.

## Materials and Methods

### Study Area and Sampling

**FIGURE 1:**
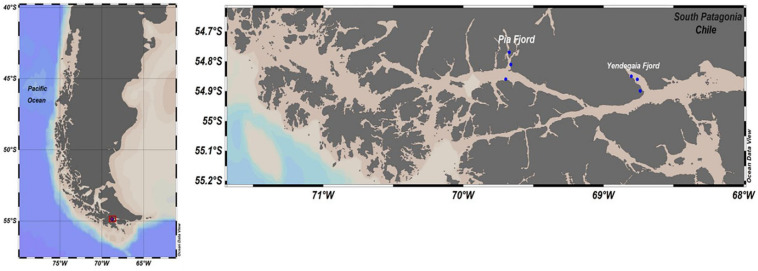
Study area and sampling sites in the Pia and Yendegaia fjords in the southern Chilean Patagonia.

Seawater samples were collected at Yendegaia fjord and Pia fjords, which are located in the southern Chilean Patagonia (Figure 1). Field campaigns were conducted during two consecutive austral winters (July 2017 and July 2018) onboard the vessel M/N Forrest. All samples were collected with a rosette system with Niskin bottles from 0, 5, 10, 25, and 50 m depth. A total of three stations per fjord were sampled along a horizontal gradient which included waters close to the glacier terminus (head) and waters outside the fjord (mouth). For microbial biodiversity analysis, 2 L of water was filtered sequentially onto 3.0 μm and 0.22 μm pore size polycarbonate membrane filters (MilliporeSigma, MA, United States) and stored in RNAlater (Thermo Fisher Scientific, MA, United States) at −80°C (liquid nitrogen) until analyzed. Hydrographic data including salinity, photosynthetically active radiation (PAR) and temperature were recorded using an SBE 25 plus and an SBE 43 CTD (Sea Bird Scientific, United States).

Samples for quantification of chlorophyll a (Chla) and dissolved inorganic nutrients were collected from 1 L seawater filtered through 0.7 μm glass fiber filters (Whatman GF/F, MilliporeSigma, MA, United States). For Chla determination, 200 ml of seawater was filtered (GF/F Whatman glass fiber filters, 0.7 μm nominal pore size) in triplicate and immediately frozen (−20°C) until later analysis via fluorometry (Turner Design TD-700), using acetone: water (90:10% v/v) for pigment extraction according to standard procedures (Parsons, 2013). Samples for nutrient analysis were filtered through GF/F filters and frozen at −20°C before analysis by spectrophotometry at the Centro de Investigación en Ecosistemas de la Patagonia (CIEP; Coyhaique, Chile) according to Strickland and Parsons method (Strickland and Parsons, 1968). To characterize dissolved organic carbon (DOC), seawater samples were taken in duplicate and filtered through 0.22 μm pore size filters (Nucleopore) into pre-combusted (450°C) glass flask and acidified with hydrochloric acid at 37%. DOC samples were analyzed on an Ol Analytical Aurora Model 1030W TOC at the Hatch Stable Isotope Laboratory (University of Ottawa, Canada) according to earlier protocol (Lalonde et al., 2014). For microbial abundances, samples (1350 mL) were taken in 2 mL cryovials, fixed with glutaraldehyde (0.1% final concentration) and stored in darkness at 80°C until laboratory analysis at the Microbial Oceanography Laboratory (Universidad de Concepción, Chile) by flow cytometry method (Marie et al., 2000). Samples for ammonium concentration were collected in triplicate and analyzed by fluorescence following earlier protocol (Holmes et al., 1999) adapted for extreme temperature ecosystems (water samples at 4°C; C. Fernández unpublished).

### DNA and RNA Extraction and Sequencing

For nucleic acid extraction, the 0.2 μm frozen filters were cut with sterilized scissors into small pieces and incubated for 45 min at 37° in 840 μL of lysis buffer (40 mmol L^–1^ EDTA, 50 mmol L^–1^ Tris hydrochloride pH 8.3 and 0.75 mol L^–1^ sucrose) with 50 μL of lysozyme solution (20 mg mL^–1^). Additionally, a second incubation with 50 μL of 20% sodium dodecyl sulfate (SDS) and 10 μL of proteinase K (20 mg mL^–1^) was completed in order to achieve cell lysis. Extraction of DNA and RNA was then performed from the lysate using an AllPrep DNA/RNA kit (Qiagen Inc., Germantown, MD, United States) following the manufacturer instructions. The quality and the quantity of the extracted DNA and RNA were measured by spectrophotometry (Thermo Scientific NanoDrop 2000). The RNA samples were reverse transcribed to cDNA with random primers using the SuperScript VILO cDNA synthesis kit (Invitrogen, Thermo Fisher Scientific, MA, United States) following the manufacturer’s protocol. For both the DNA and cDNA, the V4–V5 region of the bacterial 16s rRNA gene was amplified using universal primers 515FB-GTGYCAGCMGCCGCGGTAA and 926R-CCGYCAATTYMTTTRAGTTT (Parada et al., 2016). Amplification and paired-end sequencing (2 × 300 bp) were conducted on Illumina Miseq in the commercial laboratory Integrated Microbiome Resource (IMR, Halifax, NS, Canada) according to the protocol published earlier (Comeau et al., 2017). Sequences have been archived at the ENA under project number PRJEB40728.

### Sequence Analyses

All the reads that had a mismatch with the 16S rRNA primers contained ambiguous nucleotides (N) or were <300 bp long beyond the forward primer were removed. In addition, a stringent quality trimming criterion was applied to remove reads that had ≥10% of bases with Phred values <27. This procedure is recommended to ensure that when clustering at 97% or more, the influence of erroneous reads is minimized (Huse et al., 2010; Kunin et al., 2010). The sequences were then de-replicated and clustered at a 99% sequence similarity threshold using UCLUST (Edgar, 2010) for *de novo* OTU picking. Representative sequences were classified against the SILVA v.128 database (Quast et al., 2013). Sequence data analyses were conducted with Pyrotagger V.01 pipeline (Kunin and Hugenholtz, 2010). Sequences selected for further analysis were compared manually to the Genbank database by BLAST. Putative chimeric sequences were removed. They were identified as sequences having the best Blast alignment <90% of the trimmed read length to the reference database and >90% sequence identity to the best Blast match.

### Statistical Analyses

The OTU sequences abundance table was transformed with an Hellinger transformation (Legendre and Gallagher, 2001) with the Vegan package (Oksanen et al., 2019) in R 3.5.3 (Team, R. C. 2018). An MDS based on Bray–Curtis similarity was conducted to visualize similarities in community composition between samples with the “vegan” package. Significant differences in community structure among the different variables were tested with PERMANOVA with the adonis function. Indicator species analysis was conducted using the multipatt function of the “indicspecies” package in R (De Cáceres et al., 2012). Finally, Pearson correlation coefficient was calculated to analyze associations between abundance of indicator species and environmental variables.

## Results

### Environmental Variability

**FIGURE 2:**
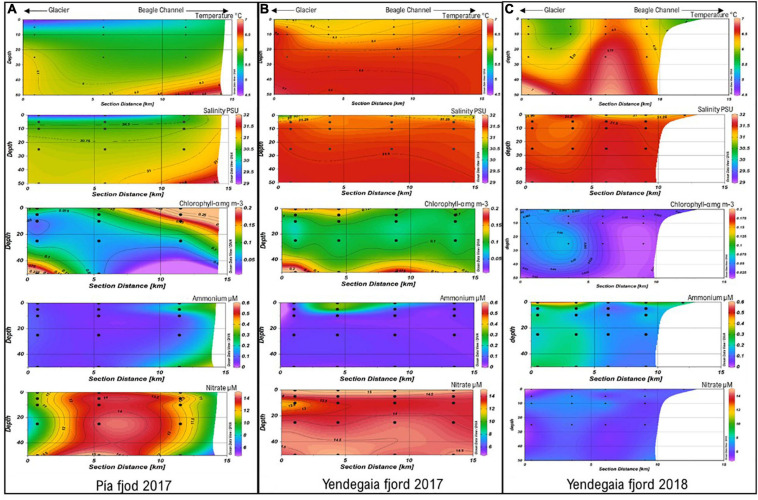
Temperature, salinity, chlorophyll a, ammonium and nitrate concentrations in the **(A)** Pia fjord July 2017 and Yendegaia fjord July 2017 **(B)** and July 2018 **(C)** from the head (glacier) to mouth (Beagle Channel) stations and across depths.

Temperature profiles in Pia fjord in 2017 were characterized by a strong vertical and horizontal gradient associated with the glacier freshwater discharge (Figure 2A). The lowest temperatures (4.5–5.0°C) were registered at the stations close to the glacier within the top 10 m of the water column (Figure 2A). Warmer waters (6.0°C) were observed at 50 m depth (Figure 2A). During 2017 presented a relatively more homogenous temperatures distribution with overall higher temperatures (6.5°C) than Pia fjord in 2017 (Figure 2B). In turn, Yendegaia in 2018 had lower temperatures associated with the glacier at the head station at 0–10 m depth, and a homogenous temperature through the water column toward the Beagle Channel (Figure 2C).

Salinity values of Pia fjord showed an influence of the glacier freshwater inputs (28–29 PSU) within the top 5 m at the head and middle stations (Figure 2A). Conversely, Yendegaia fjord showed a homogenous salinity (around 31.5 PSU), during both years (2017 and 2018), along the horizontal and vertical transects (Figures 2B,C), with an exception at the head station were salinity was 30.5 PSU at the surface in 2017 (Figure 2B).

Chlorophyll-a concentration varied from ∼ 0.06 to 0.10 mg mL^–3^ in the stations within the Pia fjord (Figure 2A). For Yendegaia, there were marked differences between 2017 and 2018 (Figures 2B,C). In 2017, the concentration of Chla ranged from 0.09 to 0.10 mg mL^–3^ across the vertical and horizontal transects (Figure 2B), whereas in 2018, concentrations were lower, varying from 0.01 to 0.07 mg mL^–3^ across the water column (Figure 2C).

Ammonium concentrations for Pia and Yendegaia fjords during 2017 were generally low (Figures 2A,B), ranging from 0.04 to 0.3 μmol L^–1^ with a maximum value (0.30 μmol L^–1^) in Yendegaia fjord at the middle station within upper 5 m (Figure 2B). In contrast, in 2018, Yendegaia fjord had higher ammonium concentrations (0.1–0.5 μmol L^–1^) with maximal values (0.5 μmol L^–1^) observed at the head station in surface waters (0 m) (Figure 2C).

Nitrate concentrations in Pia fjord were lowest (8–10 μmol L^–1^) at the head station at all the depths compared to the middle station at 50 m depth (Figure 2A). During 2017, had homogenous nitrate concentrations that ranged from 13 to 14 μmol L^–1^ (Figure 2B) across the water column. In contrast, in 2018, the nitrate concentrations decreased by half varying from 5 to 7 μmol L^–1^ (Figure 2C).

In terms of stratification of the water column, Pia fjord showed higher stratification than Yendegaia during 2017 with a surface layer (0–5 m) less saline and colder than the deeper layer (25–50 m) (Supplementary Figure 1). EWs with salinity <31 PSU (Sievers and Silva, 2008) occupied the upper 50 m of the water column. In comparison, Yendegaia 2017 had a low level of stratification with water less saline and warmer (Supplementary Figure 1). For Yendegaia 2018, the presence of Modified Sub-Antarctic Water (MSAAW; Sievers and Silva, 2008) was observed in the hole water column across all the stations (Supplementary Figure 2).

The maximum values of PAR were recorded in the surface layer of the water column (0–5 m) with highest values in Yendegaia 2017 across all stations (Supplementary Figure 3). In July 2018, Yendegaia had lower PAR values compared to 2017 (Supplementary Figure 3).

### Overall DNA and RNA Community Composition

We obtained a total of 2,722,567 16S rRNA genes and transcripts sequences of Bacteria and Archaea, from 78 samples taken during July 2017 and July 2018 in both fjords. A subset of the data was used to include only samples from which both DNA and RNA were successfully amplified (Supplementary Table 1). A total of 1,864,183 16S rRNA genes and transcripts sequences remained, divided between 44,006 different OTUs, of which 39,932 were assigned to Bacteria and 4,074 to Archaea.

**FIGURE 3:**
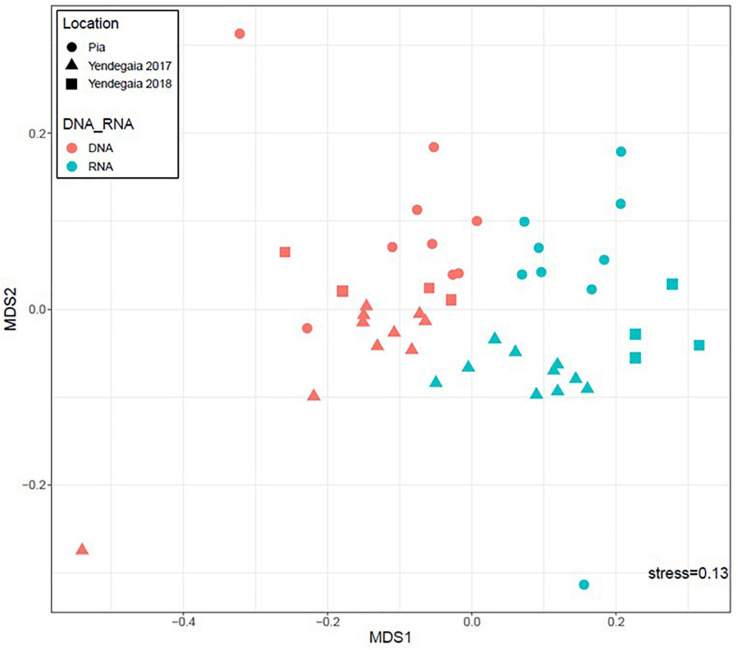
Non-metric multidimensional scaling ordination (NMDS) based on Bray–Curtis similarity analysis of microbial community composition. Colors represent DNA communities (red) and RNA communities (blue). Shapes represent the sampling sites: Pia fjord (circle), Yendegaia fjord year 2017 (triangle), and Yendegaia fjord year 2018 (square).

We compared the microbial community composition among fjords and years based on both the active fraction of the community (16S rRNA transcripts) and the total community (16S rRNA genes; Figure 3). For both fjords, the RNA fraction was different from the DNA fraction on the non-metric multidimensional scaling (NMDS) plot (PERMANOVA, *p* = 0.001, *r* = 0.10; Figure 3). Furthermore, communities from the DNA fraction were more dispersed, as seen with the higher number of outlier samples, than communities from the RNA fraction (Supplementary Figure 4). The DNA communities also had lower community diversity than the RNA fraction (Supplementary Figure 5).

### Microbial Community Composition of the Active Fraction

**FIGURE 4:**
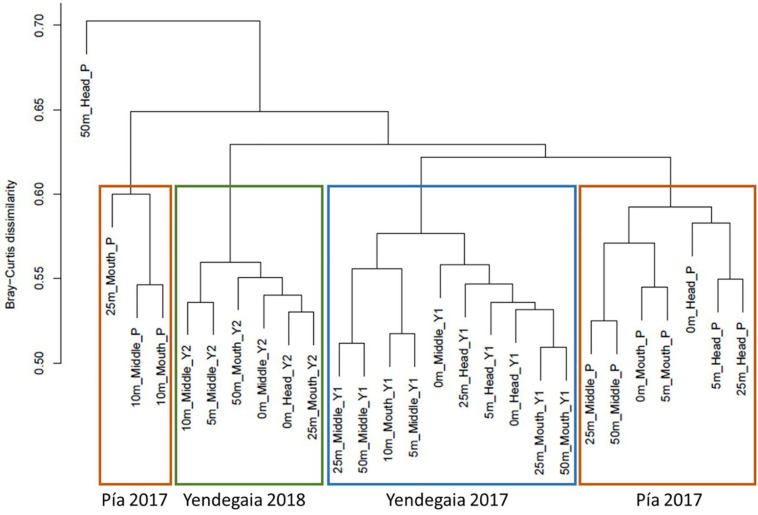
Hierarchical cluster dendrogram based on Bray–Curtis dissimilarity index showing the similarity between microbial community compositions for the RNA fraction in Pia fjord and Yendegaia fjord in 2017 and 2018.

A Bray–Curtis based dendrogram constructed from the RNA fraction revealed that microbial communities grouped according to their fjord of origin, and in the case of Yendegaia fjord, grouping also followed the sampling year (Figure 4). The PERMANOVA test based on the Bray–Curtis dissimilarity distance matrix confirmed that the microbial community of each fjord differed following their sampling site and year (*p* < 0.001, *r* = 0.18). The variable “location” better explained the differences in the microbial community composition for all the samples (Supplementary Table 2).

For Yendegaia fjord 2017 and 2018, the samples did not group according to any of the spatial variables tested (horizontal and vertical). However, for Pia fjord the clustering dendrogram grouped the samples according to a horizontal gradient from head to mouth (Figure 4), with a significant difference between the samples collected in the head of the fjord (closer to the glacier) versus the samples collected in the mouth of the fjord (PERMANOVA, *p* = 0.027, *r* = 0.14; Supplementary Table 3). No significant difference was noted for the vertical gradient (depth) in Pia fjord.

**FIGURE 5:**
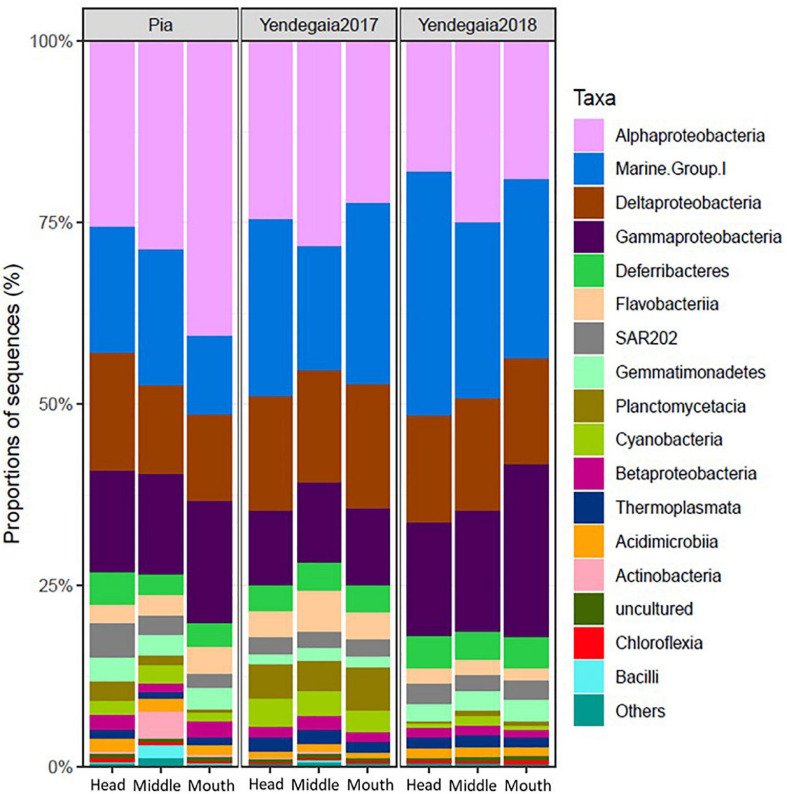
Composition of the microbial communities at the Class level in the RNA fraction in Pia fjord and Yendegaia fjord in 2017 and 2018.

Across all the samples, Proteobacteria and Thaumarchaeota represented the dominant phyla, accounting for 56 and 20% of all OTUs, respectively (Supplementary Figure 6). Some less abundant phyla were also detected in most of the samples, including Deferribacteres (3.7%), Bacteroidetes (3.4%), Chloroflexi (3%), and Planctomycetes (2.9%) (Supplementary Figure 6). At the class level, Alphaproteobacteria (24.8%), MGI (Archaea) (19.7%), Gammaproteobacteria (17%), and Deltaproteobacteria (14%) were the most abundant in the water column among fjords and years (Figure 5).

The taxonomy of the microbial community at Yendegaia fjord differed between years with a higher number of sequences of the archaea MGI Thaumarchaeota (26%) observed for the year 2018 in comparison to the year 2017 (21%; Supplementary Figure 6). Furthermore, the number of sequences for the phyla Planctomycetes and Cyanobacteria were more abundant in Yendegaia 2017 (5.4 and 3.3%, respectively) in comparison to Yendegaia 2018 (0.9 and 0.9%, respectively) (Supplementary Figure 6). Among *Proteobacteria*, the class Alphaproteobacteria were the most abundant for both years (46.6 and 38%; Figure 5). The class Deltaproteobacteria was relatively more abundant in 2017 and the class Gammaproteobacteria in 2018 (Figure 5).

Pia fjord was mainly characterized by members of the *Alpha*-, *Delta*- and *Gammaproteobacteria* with 60% of the total number of sequences, followed by *Thaumarchaeota* (14.24%) and *Bacteroidetes* (4.68%; Supplementary Figure 6). *Thaumarchaeota* were relatively more abundant at the head station compared to the inside fjord station. The classes Actinobacteria and Bacilli were only present in the Pia fjord and with more sequences at the middle station (Figure 5), mostly in the deeper samples (25–50 m).

We identified the OTU that were responsible for the significant difference observed between fjords and years with the “Indicspecies” R package. A total of 373 indicators OTUs were significant (*p* < 0.001), indicating that they were specific for one of these environments (Supplementary Figure 7). Yendegaia during 2018 hosted the highest number of indicator species (250 OTUs) and the greatest prokaryotic diversity (Supplementary Table 4). Members of the classes MGI, Gemmatimonadetes BD2-11, Deferribacteres, Thermoplasmata, Acidimicrobiia, and Cyanobacteria were indicator species present only in this fjord (Supplementary Table 4). For Pia fjord, there were 15 OTU selected as indicator species, which included members of the classes Acidobacteria and Chloroflexi-SAR202 (Supplementary Table 4). In Yendegaia fjord in 2017, 36 OTU were indicator species including members of the classes Flavobacteriia, Planctomycetacia, and Verrucomicrobiae (Supplementary Table 4). The Verrucomicrobiae class was an indicator species found only in the Yendegaia during 2017. Lastly, the phylum Proteobacteria was selected as indicator species across all the locations with a high number of different OTU sequences (173) (Supplementary Table 4).

**FIGURE 6:**
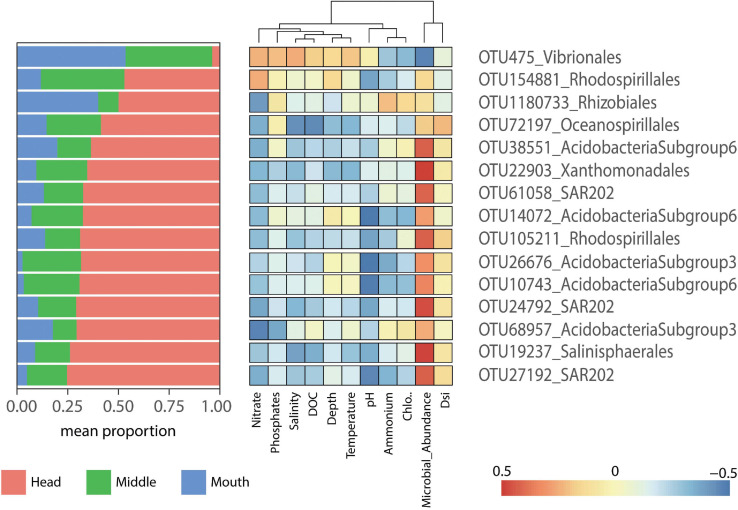
Heatmap displaying the indicator species (*p* = 0.001) for the Pia fjord. The left panel shows the mean relative contribution of the indicator species to the microbial communities by station (head, middle, and mouth). The right panel shows a heatmap of Pearson correlations between indicator species (OTU abundance) and environmental factors.

**FIGURE 7:**
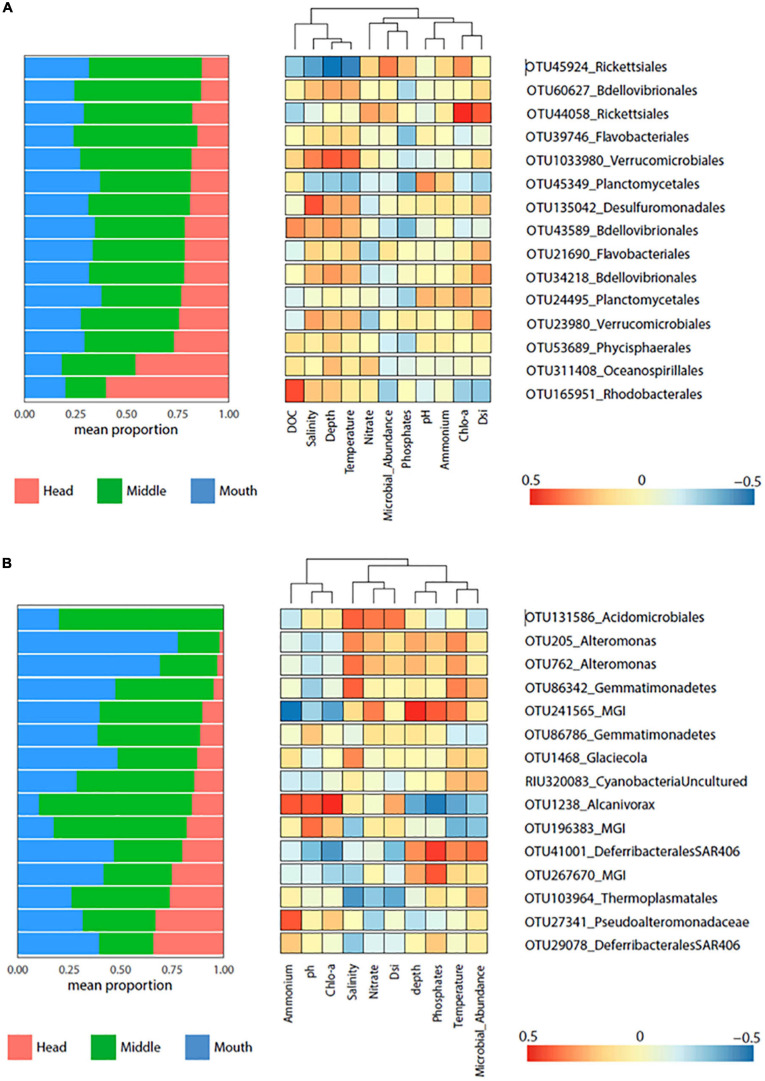
Heatmap displaying the indicator species (*p* = 0.001) for Yendegaia fjord 2017 **(A)** and 2018 **(B)**. Left panel shows the mean relative contribution of the indicator species to microbial community detected by stations (head, middle, and mouth). Right panel shows heatmap of Pearson correlation between indicator species (OTU abundance) and environmental factors.

Correlations between indicator species and the environmental variables were determined for each fjord. In general, there were no significant correlations between indicator species and environmental variables in each fjord, except for the positive correlation between Nitrate and OTU105211_*Rhodospirillales* for the Pia fjord at the head station (*p* = 0.05) (Figure 6), and the negative correlation for the OTU1238_*Alcanivorax* and temperature in Yendegaia during 2018 at the middle station (*p* = 0.01) (Figure 7).

The major proportion of indicator species selected for Pia fjord were found at the head station except for the OTU475_*Vibrionales*, which was more present at the mouth station (Figure 6). OTU475_*Vibrionales* show a pattern of correlation different to the rest of the OTU indicators, with a positive correlation with nitrate, phosphate, and salinity and a negative correlation with microbial abundance (Figure 6). In contrast, most of the other OTU indicators in Pia fjord (11 OTU) were negatively correlated with nitrate and positively with microbial abundance (Figure 6). The OTUs *Acidobacteria* subgroup 6-3 (14,072, 26,676, and 10,743) and the *Chloroflexi*-SAR202 (OTU 27192) were negatively correlated with pH, and the *Oceanospirillales* (OTU72197) was negatively correlated with phosphate and salinity.

For Yendegaia fjord in 2017, 15 OTUs indicators were selected based on their abundance. In general, the OTUs showed to be more abundant at the middle station, except the OTU311408 (*Oceanospirillales*) and OTU165951 (*Rhodobacterales*) more abundant at the head station (Figure 7). The environmental variables temperature, depth, and salinity were the variables that showed a better pattern of correlation with 5 OTUs (Figure 7). A positive correlation was observed for the OTU43589/OTU60627 (*Bdellovibrionales*), OTU135042 (*Desulfuromonadales*) and OTU1033980 (*Verrucomicrobiales*), while the OTU45924 (*Rickettsiales*) and OTU45349 (*Plantomycetales*) correlated negatively (Figure 7). Finally, strong positives correlations were observed for the OTU165951 (*Rhodobacterales*) and DOC and OTU44058 (*Rickettsiales*) with Chla and Dsi (Figure 7).

For Yendegaia fjord 2018, 15 OTUs indicators were selected with the same criteria as in Yendegaia 2017 fjord. The selected OTUs were mostly present at the stations middle and mouth (Figure 7). The indicator species *Alteromonas* (OTU205/762), *Glaciecola* (OTU1468), and *Gemmatimonadetes* (OTU86342), which were relatively more abundant at the mouth station, were positively correlated with salinity and temperature (Figure 7). The OTU131586 (*Acidomicrobiales*) and OTU103964 (*Thermoplasmatales*), both relatively more abundant at the middle station, showed a similar pattern of correlation but in an inverse manner, with *Acidomicrobiales* positively correlated with salinity, nitrate, and Dsi (Figure 7). Furthermore, the OTU241565 (MGI) and OTU41001 (*Defirrebacterales*-SAR406) exhibited a positive correlation with temperature, phosphate, and depth, meanwhile both had a negative correlation with Chla, pH, and ammonium. Conversely, the OTU1238 (*Alcanivorax*) correlated with the same set of variables previously described, but in the opposite way (Figure 7). Finally, the OTU 27341 (*Pseudoalteromonadaceae*), with a similar proportion of sequences across the stations, presented positive correlation ammonium (Figure 7).

### Comparison Between Total (rDNA) and Active (rRNA) Community Composition

In order to compare more precisely the rRNA and rDNA community composition at the OTU level, we computed the Bray–Curtis index between pairs of DNA and RNA samples. The results showed that the higher dissimilarity between fractions was observed in Yendegaia 2018 fjord (Supplementary Figure 8). The median Bray–Curtis values were the same for Yendegaia 2017 and Pia fjords, however Pia fjord had more variations between samples. We then looked at the community composition between fractions to identify possible differences (Supplementary Figure 9). For Yendegaia 2018 fjord, the difference was due to a large proportion of sequences associated to the class Bacilli and Negativicutes (both phylum Firmicutes) present in the DNA fraction and absent in the RNA (Supplementary Figure 9). In Yendegaia 2017 and Pia fjords, the DNA fraction presented generally higher proportions of *Flavobacteriia* (phylum Bacteroidetes), while higher proportions of *Deltaproteobacteria* were observed in the RNA fractions. In Yendegaia 2017 fjord, MGII Archaea were also present in the DNA fraction.

## Discussion

### I-Differences Between Fjords

The characterization of the microbial communities of two contrasting Patagonian fjords revealed that each harbored a specific community. Most of the indicator species for Pia fjord were found at the head station toward the glacier terminus. They comprised OTUs belonging to *Alphaproteobacteria*, abundant in fjord with mixed layers, and *Acidobacteria*, typical members of freshwater systems (Piquet et al., 2010; Fortunato et al., 2013). It highlights the hybrid nature of these fjords between terrestrial and marine environments (González et al., 2013) where prokaryotic communities from different origins may coexist pending on their phylogenetic and metabolic requirements. Members from the classes Actinobacteria and Bacilli (*Firmicutes*) were only detected in Pia fjord and with a more significant proportion in the station inside the fjord (Figure 5). Studies across salinity gradients evidenced that phyla Actinobacteria and Firmicutes decrease with increasing salinity (Piquet et al., 2010; Fortunato and Crump, 2015), which is consistent with our observations. Interestingly, members of the SAR202 clade were also more present in Pia fjord compared to Yendegaia 2017. This cluster is usually found in deep ocean waters below 200 m (Mehrshad et al., 2018), and described as sulfite-oxidizers with an essential role in the sulfur turnover in the dark ocean (Mehrshad et al., 2018). Its presence as one of the most abundant taxa and as an indicator species in our surface water data set provides new information on the distribution of this group and its potential role in sulfur biogeochemistry in Patagonian fjords where it has not been previously reported.

In Yendegaia fjord during 2017, the number of sequences for the classes Planctomycetia, Cyanobacteria, and Thermoplasmata were higher than in Pia fjord (Figure 5). A recent study reported that members of the phylum Planctomycetes could fix nitrogen in surface waters of the Pacific and Atlantic oceans where the limitation of iron and nitrogen could favor these diazotrophs (Delmont et al., 2018). Our finding shows that these three classes doubled their sequence proportion in Yendegaia compare to Pia fjord, suggesting that Yendegaia environmental conditions are more suitable for the development of nitrogen fixation and photosynthetic microorganism. For *Archaea*, most of the sequences belonged to the Marine group II (MGII), which has earlier been shown to have contrasted abundance and distribution in polar waters (Galand et al., 2006, 2008). Church et al. (2003) reported that MGII abundance was low along the entire water column and that it did not follow seasonal or horizontal patterns in the Southern Ocean. However, in the coastal Arctic Ocean, MGII was reported as the most common archaeal group in surface water samples (Galand et al., 2006, 2008). Furthermore, it was suggested that the presence of MGII along coastal waters could be linked to river waters containing high concentrations of particulate matter (Wells et al., 2006; Galand et al., 2008). It could explain the higher proportion of MGII observed in Yendegaia fjord, which receives waters from a proglacial river with a high input of particulate matter.

For Yendegaia 2017, the indicator species were dominated by members of the phyla Planctomycetes, Bacteroidetes (*Flavobacteriia*), and Proteobacteria (*Deltaproteobacteria*). *Deltaproteobacteria* is an important member of bacterial communities of marine sediments which has been related to sulfur cycle (Zeng et al., 2009), while *Planctomycetes* is associated to nitrogen fixation in environments with iron and nitrogen limitation (Delmont et al., 2018). *Flavobacteriia* has been found to correlate positively with Chla in the Southern Ocean (Abell and Bowman, 2005). Finally, OTUs from the class Verrucomicrobia a common member of marine microbial communities with global distribution was also selected as an indicator species in Yendegaia fjord.

The differences in community composition between the two fjords are possibly related to the morphological and hydrographical conditions of the fjords. Besides the salinity gradient, the Pia fjord is characterized by the presence of a pronounced sill at the outer part of the fjord (SHOA, Hydrographic and Oceanographic Service of the Chilean Navy), which limits the inflow of Subantarctic water (SAAW) into the fjord and increase the residence time of EW. Moreover, in Pia, water turbidity is high along the whole fjord and the entire water column, as opposed to Yendegaia where the inorganic mater input and turbidity is only observed in the upper 20 m (Giesecke et al., 2019). The upwelling in Pia fjord produced by the discharge of freshwater from the marine-terminating glacier could favor the appearance of microorganisms from deep layers of water such as SAR202. Thus, differences in the morphological characteristic, sediment load and salinity/temperature could be responsible for the observed differences in microbial communities between both fjords.

Finally, there were also some common microbial features between the fjords. Consistent with previous studies in high latitude environments, members of the *Alpha-, Gamma-*, and *Deltaproteobacteria* were the most abundant classes in the entire water column accounting for 56% of the sequences present in the active fraction for both fjords (Zeng et al., 2009; Teske et al., 2011; Gutiérrez et al., 2015, 2018; Valdés et al., 2017). Concerning *Archaea*, members of the *Thaumarchaeota* (MGI) (20% of the total sequences across the whole set of samples) have been described as a major contributor to oceanic microbial diversity, but their presence varies according to depth and environments (Santoro et al., 2019). In high latitude environments, higher proportions of MGI sequences have been found in surface water layers (Bano et al., 2004; Galand et al., 2008; Gutiérrez et al., 2015, 2018) compare to subtropical ecosystems where MGI are found commonly in deep layers (Agogue et al., 2008; Church et al., 2010). Interestingly, no vertical community changes, on the total or active fraction of the microbial communities, were observed according to depth in the water column of both fjords and years. It probably reflects the fact that during winter, the water column of fjord tends to be vertically homogenous principally due to the low input of freshwater and the strong wind mixing (Bianchi et al., 2020). However, in Pia fjord, a clear horizontal community change was observed in the active fraction from the head to the mouth of the fjord. Changes in the proportion of the sequences of phyla Thaumarchaeota and Planctomycetacia as well as Gammaproteobacteria and Alphaproteobacteria were recorded. The water column inside the Pia fjord was colder and less saline than at the mouth (Figure 2), which has been reported as possible drives for changes at the class level in estuarine and coastal saline gradients (Fortunato et al., 2012; Fortunato and Crump, 2015; Herlemann et al., 2016).

### II-Differences Between Years

In Yendegaia fjord the prokaryotic community composition was compared between July 2017 and July 2018, and significant differences were observed. In 2018 there were more sequences belonging to MGI *Thaumarchaeota*, *Gemmatimonadetes*, and *Gammaproteobacteria*, while in 2017, *Flavobacteria*, *Planctomycetacia*, and *Cyanobacteria* were relatively more abundant. The differences are possibly explained by changes in the physicochemical characteristics of the water column and could be a result of climatological differences between years.

During July 2017, the upper 50 m of the water column showed evidence of stratification in comparison to July 2018. Moreover, the levels of PAR recorded in the fjord were higher for July 2017 compared to July 2018. The decrease in the PAR index during July 2018 is consistent with the decrease of Chla in the water column. Stratified water column and a high PAR index could favor low-size autotrophic cells such as *Cyanobacteria* (Cuevas et al., 2019). In the same way, the significant presence of *Flavobacteria* (phylum Bacteroidetes) during July 2017 could be related to a possible phytoplankton bloom related to the PAR levels and nitrate concentrations. *Flavobacteria* usually do not present a distribution pattern directly linked to physical or chemical environmental variables (Gomez-Pereira et al., 2010), but they seem associated to phytoplankton blooms because of their ability to degrade associated molecules (Kirchman, 2002). Conversely, the increase observed for MGI *Thaumarchaeota* in 2018 could be related to the decline in the PAR index and the increase of ammonium during July 2018. The presence of MGI in surface layers of high latitude environments has been related to low solar radiation intensity since MGI is sensitive to reactive oxygen species (ROS) generated in the biological and photochemical process (Merbt et al., 2012; Luo et al., 2014; Pedneault et al., 2014; Tolar et al., 2016). Additionally, MGI is an ammonia oxidizer archaeon with high substrate affinity (Lehtovirta-Morley, 2018), which allows it to proliferate rapidly when ammonia concentrations increase. Furthermore, a high proportion of the OTUs indicator species for July 2018 was related to the non-photosynthetically activity such as OTU1238 (*Alcanivorax/Oceanospirillaceae*), OTU41001/29078 (SAR406), OTU27341 (*Pseudoalteromonadaceae*), MGI and *Thermoplasmatales* OTUs, among others. Some of these species, such as SAR406, *Pseudoalteromonadaceae* and *Oceanospirillaceae*, are described to participate in sulfur and nitrogen biogeochemical cycles (Wright et al., 2014; Delmont et al., 2018).

Finally, in our dataset, the rDNA community did not reveal the clear differences seen with rRNA data. The fact that the RNA fraction presented greater diversity, as well as less dispersion and stronger biogeographical patterns, evidence that rRNA is a useful tool to detect the response of microorganisms to different environmental conditions. In addition, we noted the presence of *Bacilli* and *Negativicutes* in some DNA samples. These classes are known to be associated to humans rather marine plankton, and could therefore originate from contamination. The fact that they were a minor component of the RNA fraction, is an additional argument for the usefulness of RNA in environmental studies. It must be mentioned, however, that the use of rRNA as an indicator of growing or active microorganism is controversial and it should be used as an indicator of potential activity rather than actual activity (Blazewicz et al., 2013).

## Conclusion

In conclusion, our study shows that differences in the hydrographic properties of the water column, due to the hydro-morphological characteristics of the fjords, can influence the structure of the prokaryotic community on the first 50 m of the water column. In Pia fjord, the prokaryotic community was influenced from above (melt freshwater) and below (upwelled seawater) due to the presence of the marine-terminating glacier. Meanwhile, in Yendegaia, which has a land-terminating glacier, the prokaryotic assemblage was similar to those generally described for oceanic waters. In addition, major inter-annual taxonomic changes in Yendegaia fjord could be attributable to an increase of PAR that would promote the development of small size photosynthetic microorganisms leading to significant difference in prokaryotic community composition. Yendegaia could represent a typical fjord under future climate predictions, since Aquatic Critical Zones (ACZs) (Bianchi et al., 2020) reported that fjords are expected to experience substantial ice melting and glacial retreat (IPCC, 2019), which will modify the hydrographic properties, nutrients, light availability, and climatologic conditions of this zone (Iriarte et al., 2018). The glacier associated to Yendegaia fjord has indeed already retreated by 12 km from its origin. This retreat has impacted the physical, chemical, and biological properties of the fjord generating a state of low primary production (Meire et al., 2017; Giesecke et al., 2019). With this study we add data to the current knowledge on the impact of fjord glacier melting by showing that it favored microorganisms such as nitrogen-fixing *Planctomycetes*. Alterations in the structure of the usual microbial community and subsequent changes in the trophic chain (Langenheder et al., 2003; Gutiérrez et al., 2015) will affect the basic functionality of future fjords ecosystems.

## Data Availability Statement

The names of the repository/repositories and accession number(s) can be found in the article/see section Materials and Methods.

## Author Contributions

CM-M, CF, HG, and PG designed the experiment. CM-M performed the experiment. CM-M and PG analyzed the data and wrote the manuscript. All authors reviewed and approved the manuscript.

## Conflict of Interest

The authors declare that the research was conducted in the absence of any commercial or financial relationships that could be construed as a potential conflict of interest.
