# Supplementary material for: Different Active Microbial Communities in Two Contrasted Subantarctic Fjords
Source: Front Microbiol. 2021 Jun 24;12:620220. doi: 10.3389/fmicb.2021.620220 (PMC8264266; doi:10.3389/fmicb.2021.620220)
Supplement: Supplementary file 1 [file Data_Sheet_1.docx]

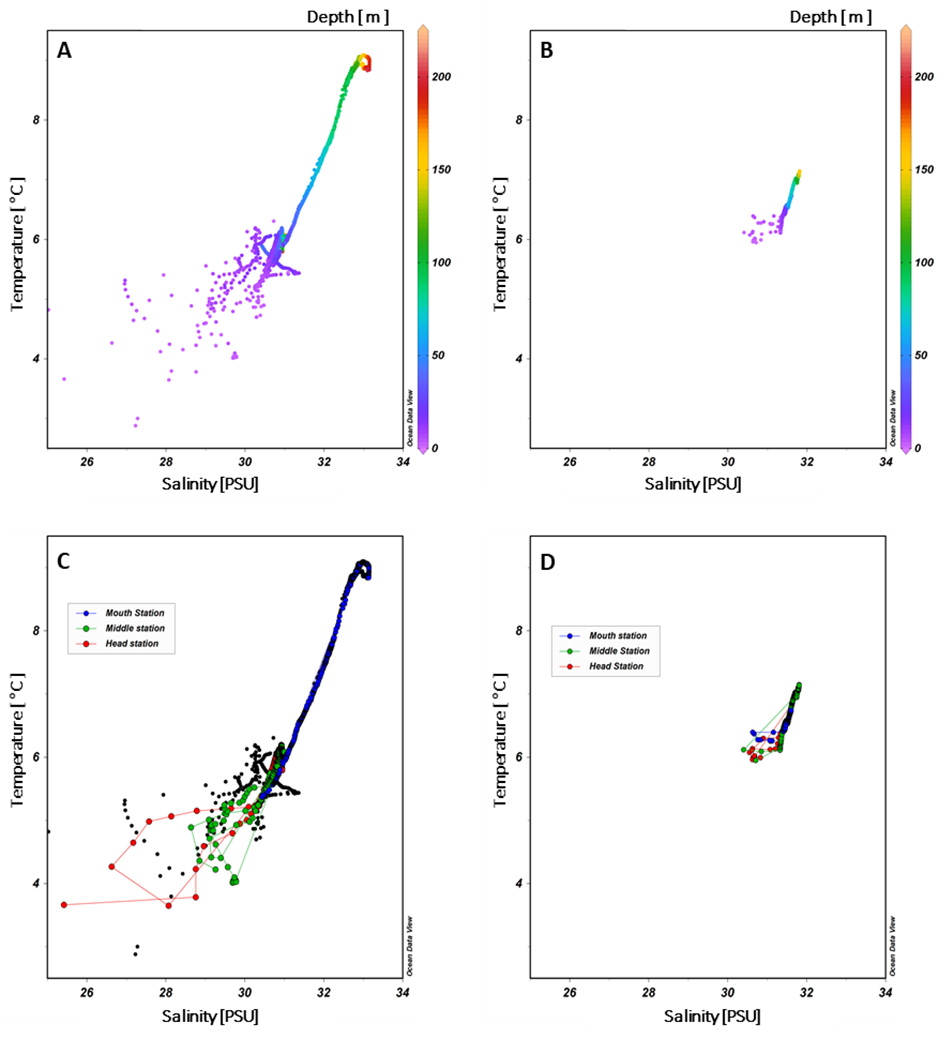
 Supplementary Figure 1. Temperature-salinity diagram for the Pia Fjord (left) and the Yendegaia Fjord (right) in 2017.


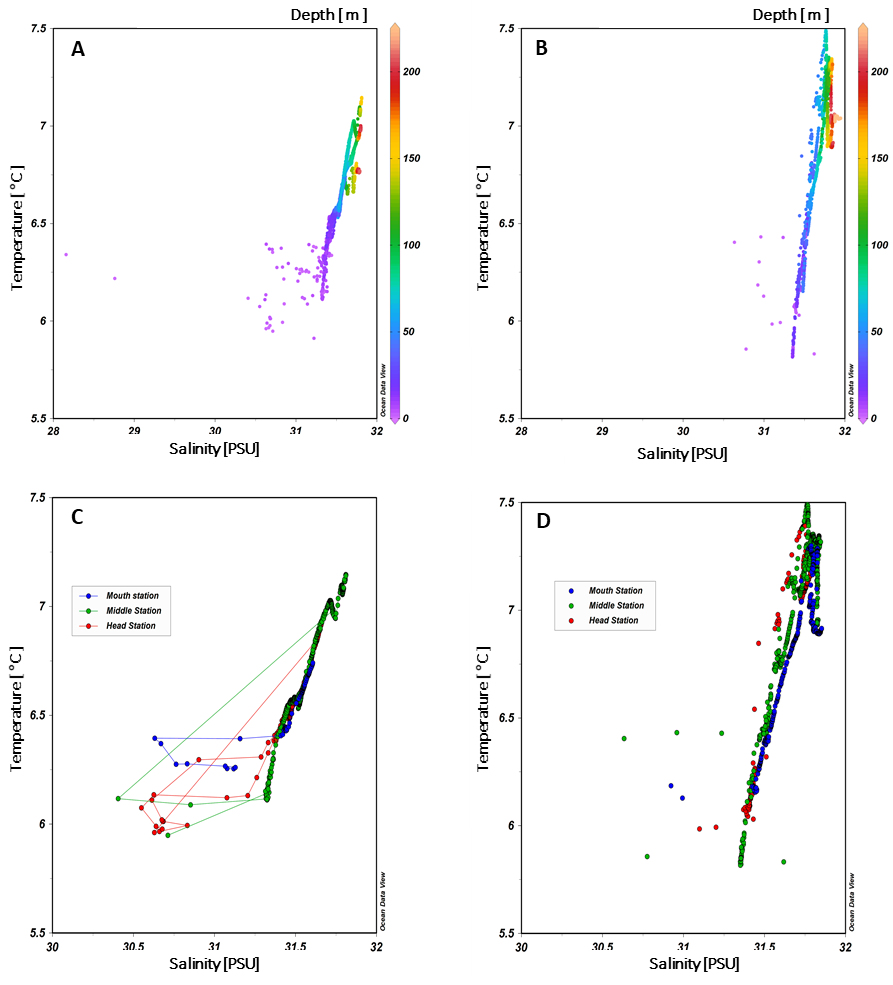
 Supplementary Figure 2. Temperature-salinity diagram for the Yendegaia Fjord in 2017 (left) and the Yendegaia Fjord in 2018 (right).


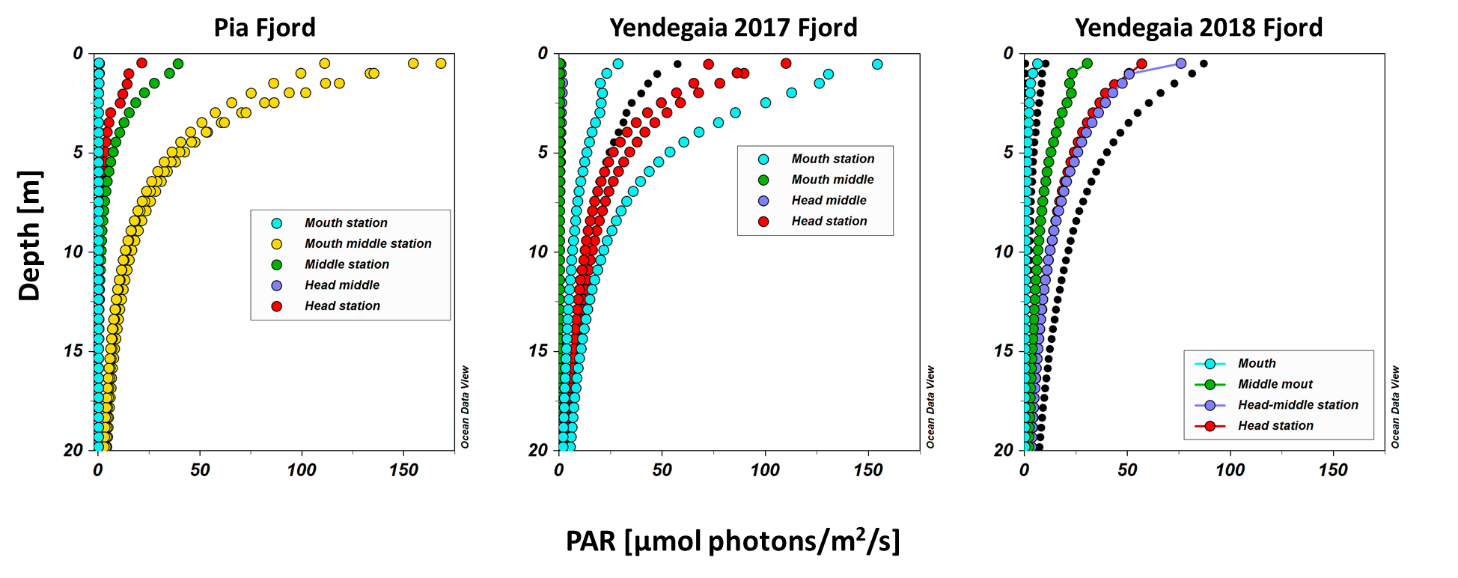
 Supplementary Figure 3. Photosynthetically Active Radiation (PAR) from 0 to 20 m depth in each sampling location in the Pia and Yendegaia fjords. The colours represent the different stations.


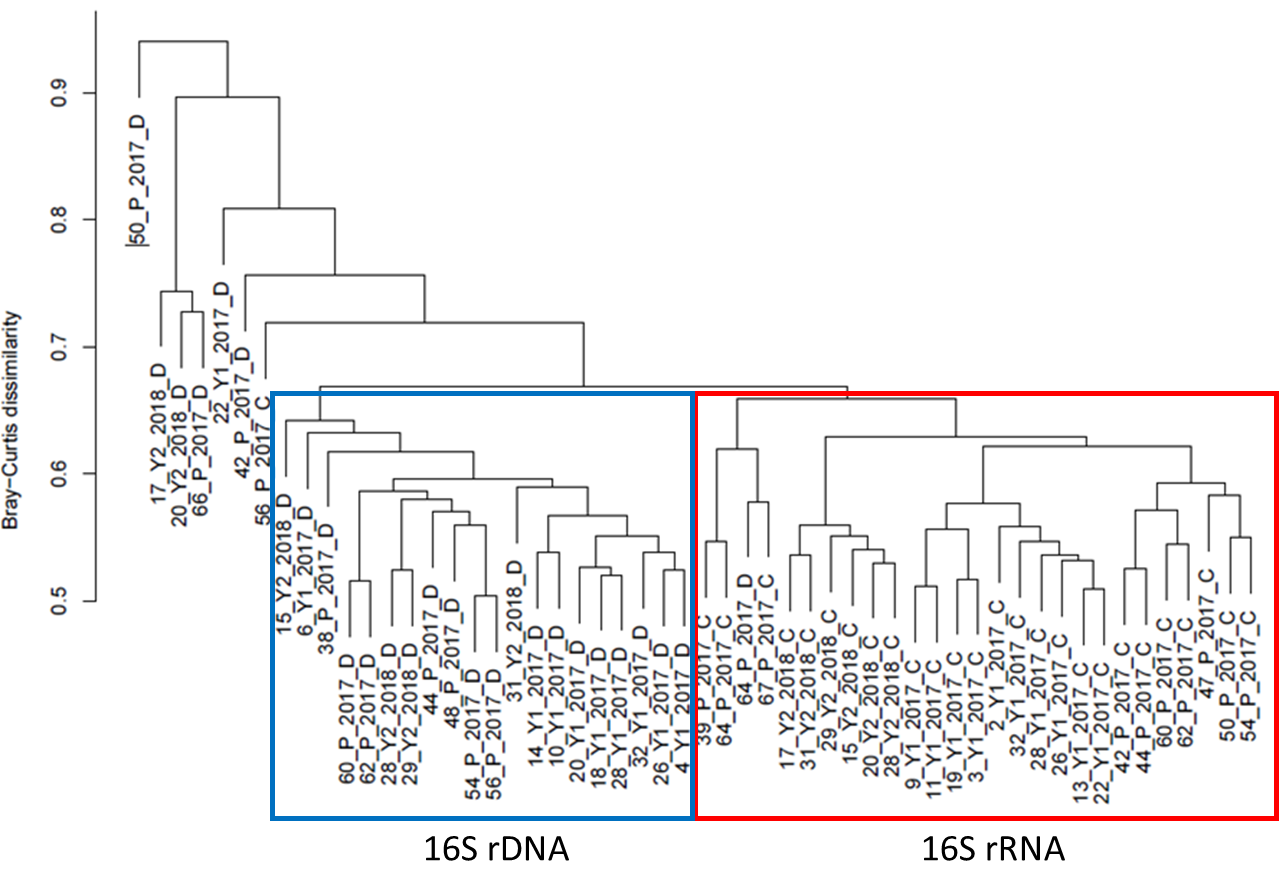
 Supplementary Figure 4. Hierarchical cluster dendrogram based on Bray-Curtis dissimilarity index showing the similarity between microbial community compositions for the DNA and RNA fraction in both fjords and years.


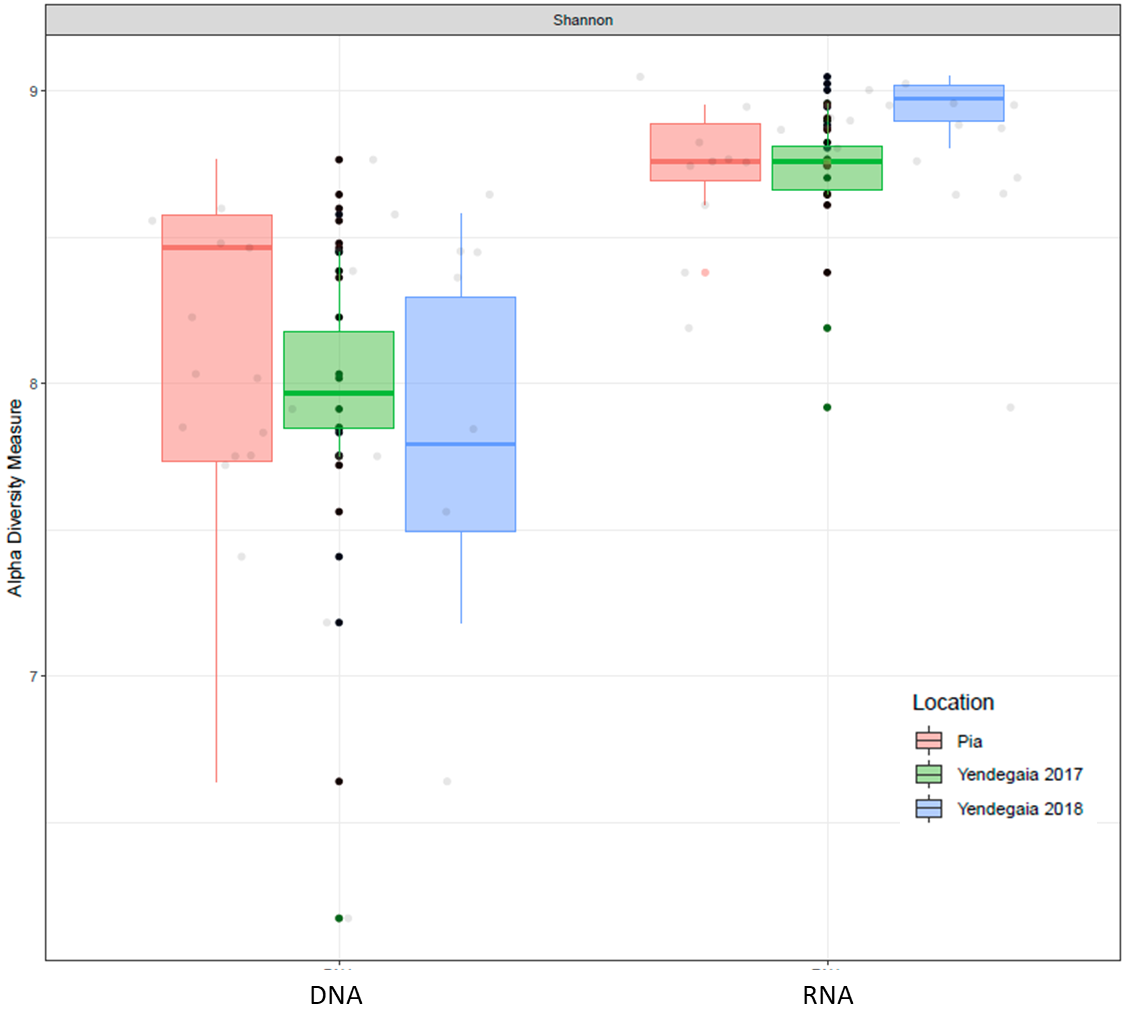


Supplementary Figure 5. Boxplot based on Shannon diversity index showing microbial community α-diversity for the DNA and RNA fraction in both fjords and years.


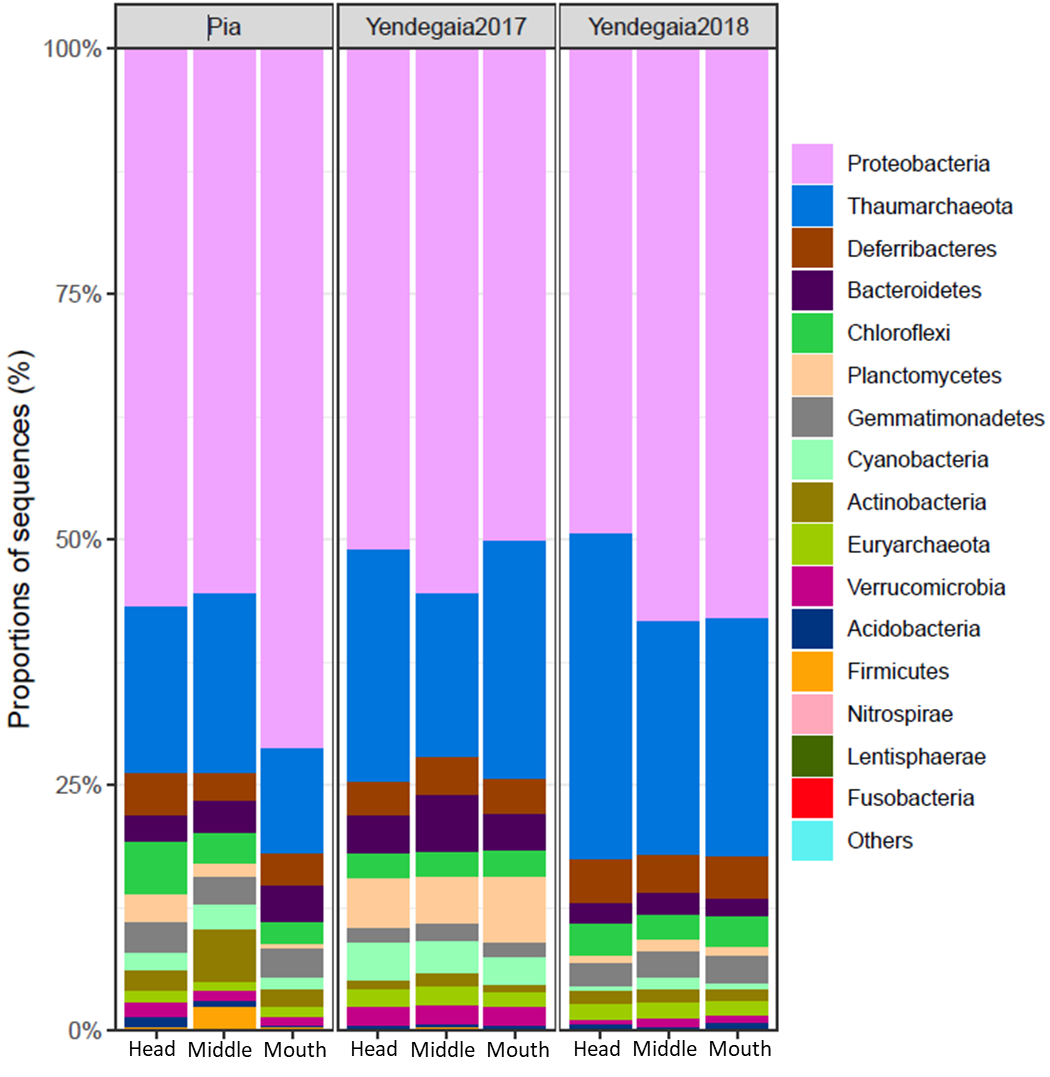
 Supplementary Figure 6. Relative proportion of 16S rRNA prokaryotic sequences at the Phylum level in the RNA fraction in the Pia fjord and the Yendegaia fjord.


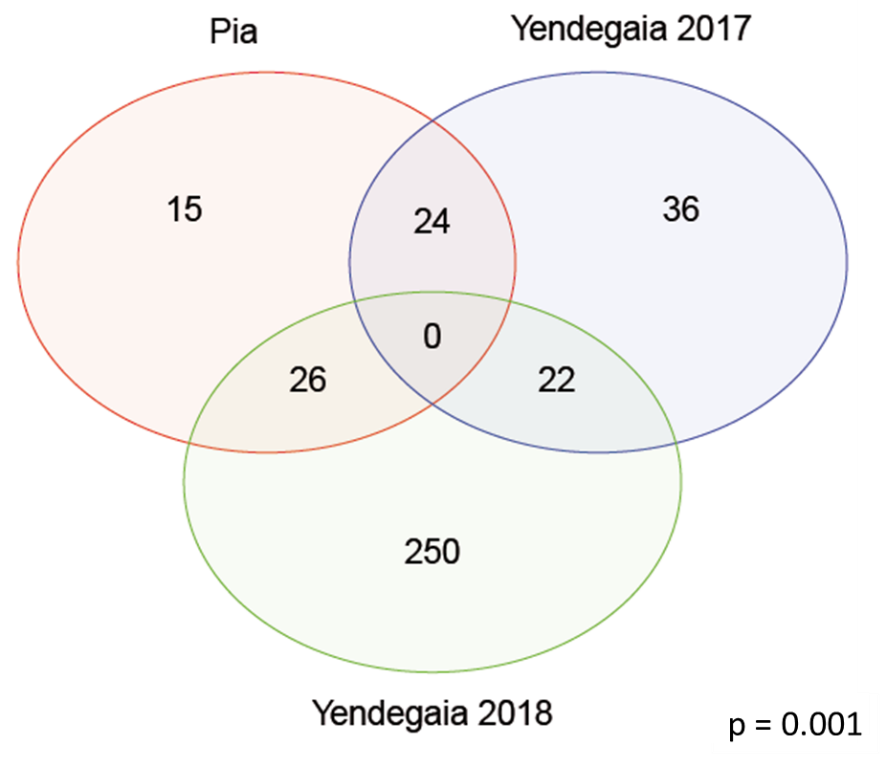


Supplementary Figure 7. Venn diagram showing the number of indicator prokaryotic taxa that are exclusive or shared among the different sampling sites.

**
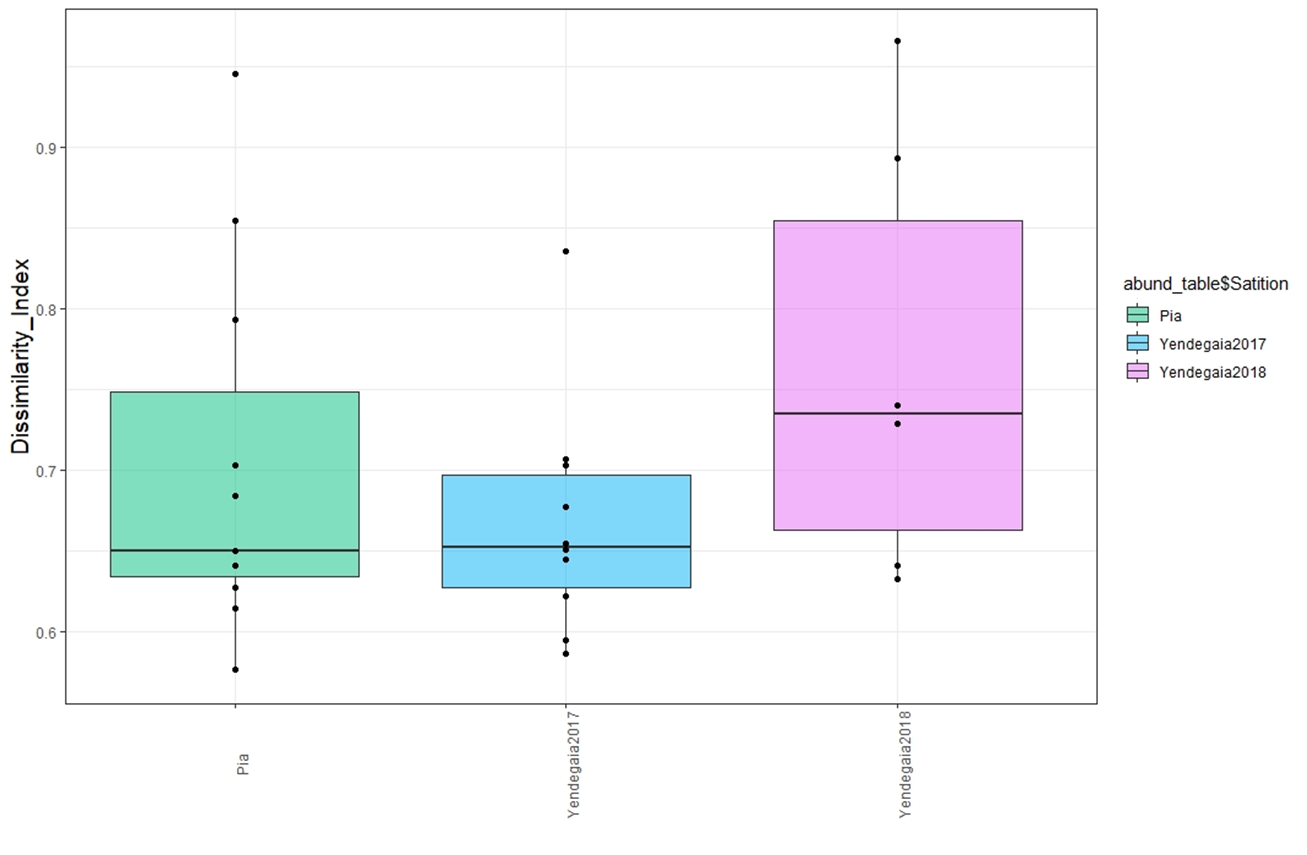
**

#### Supplementary figure 8: Bray-Curtis dissimilarity between the DNA and RNA fractions for microbial communities sampled. Only samples for which both the DNA and RNA fractions were successfully amplified were considered for this analysis.


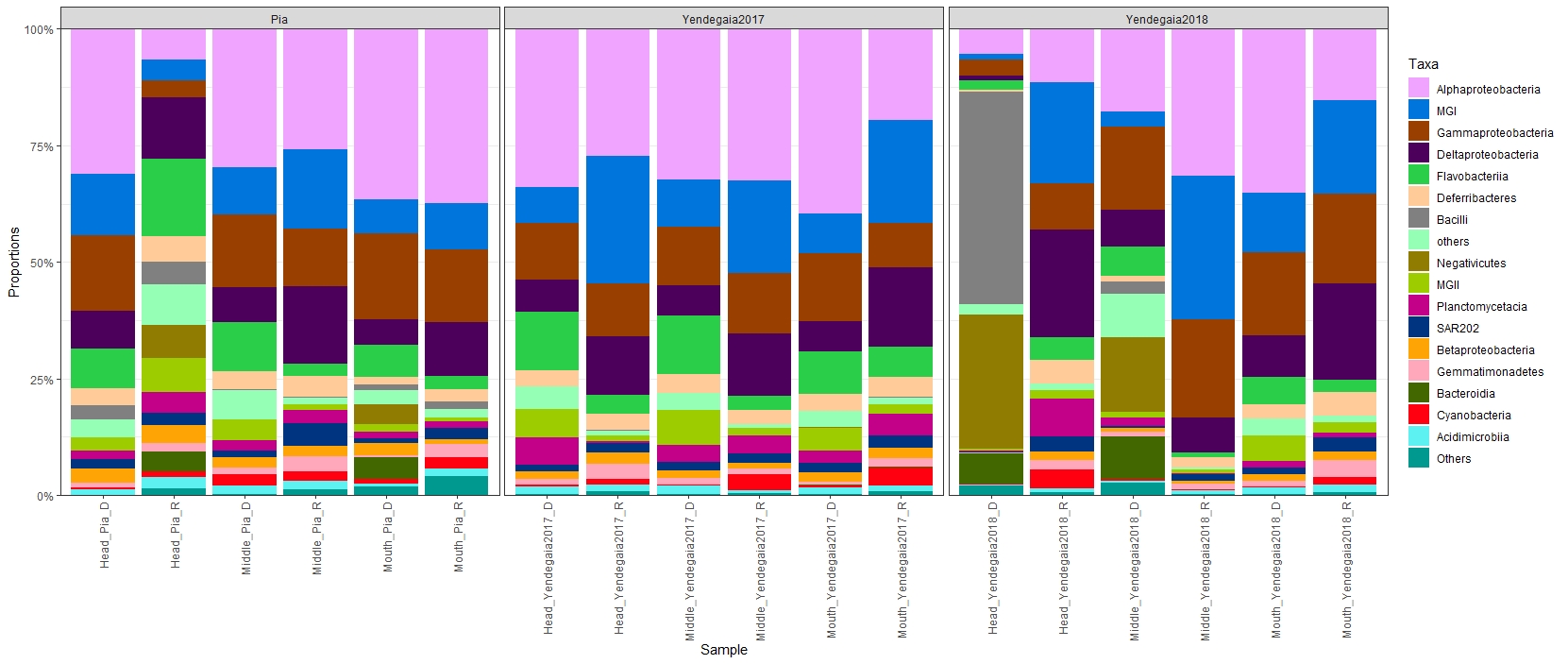


Supplementary Figure 9: Relative proportion of 16S rRNA prokaryotic sequences at the Phylum level in the DNA vs RNA fraction in the Pia fjord and the Yendegaia fjord. D denotes DNA; R denotes RNA.
