# Supplementary material for: Different Active Microbial Communities in Two Contrasted Subantarctic Fjords
Source: Front Microbiol. 2021 Jun 24;12:620220. doi: 10.3389/fmicb.2021.620220 (PMC8264266; doi:10.3389/fmicb.2021.620220)
Supplement: Supplementary file 2 [file Table_1.DOCX]

Supplementary Table 1. Number of sequences detected for prokaryotic assemblages in water samples from Pia (P), Yendegaia 2017 (Y1) and Yendegaia 2018 (Y2) fjords.

| **Sample** | **Total number of reads** | **DNA reads** | **RNA reads** |
| --- | --- | --- | --- |
| 0m_Head_P | 68040 | 26120 | 41920 |
| 5m_Head_P | 60523 | 19455 | 41068 |
| 25m_Head_P | 74677 | 45615 | 29062 |
| 50m_Head_P | 79699 | 48293 | 31406 |
| 10m_Middle_P | 60160 | 15346 | 44814 |
| 25m_Middle_P | 89904 | 41320 | 48584 |
| 50m_Middle_P | 74049 | 24414 | 49635 |
| 0m_Mouth_P | 97325 | 48318 | 49007 |
| 5m_Mouth_P | 96352 | 50878 | 45474 |
| 10m_Mouth_P | 77071 | 32150 | 44921 |
| 25m_Mouth_P | 59150 | 31318 | 27832 |
| **Total Reads Pia** | **836950** | **383227** | **453723** |
| 0m_Head_Y1 | 56624 | 20665 | 35959 |
| 5m_Head_Y1 | 63363 | 28757 | 34606 |
| 25m_Head_Y1 | 59541 | 30225 | 29316 |
| 0m_Middle_Y1 | 48446 | 16479 | 31967 |
| 5m_Middle_Y1 | 50702 | 28310 | 22392 |
| 25m_Middle_Y1 | 64841 | 24701 | 40140 |
| 50m_Middle_Y1 | 83960 | 41670 | 42290 |
| 10m_Mouth_Y1 | 37592 | 19836 | 17756 |
| 25m_Mouth_Y1 | 48709 | 3949 | 44760 |
| 50m_Mouth_Y1 | 70693 | 24955 | 45738 |
| **Total Reads Y1** | **584471** | **239547** | **344924** |
| 0m_Head_Y2 | 76878 | 26231 | 50647 |
| 0m_Middle_Y2 | 57404 | 15269 | 42135 |
| 5m_Middle_Y2 | 63522 | 23056 | 40466 |
| 10m_Middle_Y2 | 96543 | 53161 | 43382 |
| 25m_Mouth_Y2 | 65242 | 31722 | 33520 |
| 50m_Mouth_Y2 | 83173 | 39231 | 43942 |
| **Total Reads Y2** | **442762** | **188670** | **254092** |

Supplementary Table 2. Permutational Multivariate Analysis of Variance (PERMANOVA) testing the effect of the factors “location” (Pia, Yendegaia2017, Yendegaia2018), Depth (0-50 m) and Station (head, middle, mouth) on the RNA fraction of the prokaryotic communities. Key to abbreviations and column headings: D.f: degrees of freedom; MS: mean square; F: F ratio; R^2^: coefficient of determination; P: p-value. Significant results are in bold.

|  | Df | MS | F | R2 | P |
| --- | --- | --- | --- | --- | --- |
| Depth | 1 | 0.1959 | 0.19592 | 0.03878 | 0.157 |
| Station | 2 | 0.3931 | 0.19653 | 0.0778 | 0.084 |
| Location | 2 | 0.9229 | 0.46144 | 0.18266 | ***0.001*** |

Supplementary Table 3. Permutational Multivariate Analysis of Variance (PERMANOVA) testing the effects of the variables Depth (0-50 m) and Station (head, middle, mouth) on the composition of the active fraction of the prokaryotic communities in each of the locations. Key to abbreviations and column headings: D.f: degrees of freedom; MS: mean square; F: F ratio; R^2^: coefficient of determination; P: p-value. Significant results are bold

| Location |  | Df | MS | F | R2 | P |
| --- | --- | --- | --- | --- | --- | --- |
| Yendegaia 2017 | Depth | 1 | 0.17192 | 1.1286 | 0.12103 | 0.141 |
|  | Station | 2 | 0.1673 | 1.0983 | 0.23556 | 0.178 |
| Yendegaia 2018 | Depth | 1 | 0.15031 | 0.95867 | 0.19743 | 0.7306 |
|  | Station | 2 | 0.14872 | 0.94851 | 0.39068 | 0.8278 |
| Pia 2017 | Depth | 1 | 0.21996 | 1.1964 | 0.11185 | 0.135 |
|  | Station | 2 | 0.27582 | 1.5002 | 0.14025 | ***0.027*** |

Supplementary Table 4. Distribution of indicator microbial taxa among the fjords (p = 0.001).

| **Location** | **N° bacterial**  **taxa** | **N° Indicator**  **microbial taxa** | **N°**  **OTU** | **Microbial taxa** | |
| --- | --- | --- | --- | --- | --- |
|  |  |  |  | **Class/Order Level** | |
| Pia 2017 | 6600 | 15 | 1 | *Gammaproteobacteria* | *Vibrionales* |
|  |  |  | 1 | *Gammaproteobacteria* | *Salinisphaerales* |
|  |  |  | 1 | *Gammaproteobacteria* | *Xanthomonadales* |
|  |  |  | 1 | *Gammaproteobacteria* | *Oceanospirillales* |
|  |  |  | 3 | *Acidobacteria* | *Subgroup 6* |
|  |  |  | 2 | *Acidobacteria* | *Subgroup 3* |
|  |  |  | 2 | *Alphaproteobacteria* | *Rhodospirillales* |
|  |  |  | 1 | *Alphaproteobacteria* | *Rhizobiales* |
|  |  |  | 3 | *Chloroflexi -SAR202* |  |
| Yendegaia 2017 | 3499 | 36 | 3 | *Alphaproteobacteria* | *Rickettsiales* |
|  |  |  | 1 | *Alphaproteobacteria* | *Rhodobacterales* |
|  |  |  | 5 | *Deltaproteobacteria* | *Bdellovibrionales* |
|  |  |  | 1 | *Deltaproteobacteria* | *Desulfuromonadales* |
|  |  |  | 3 | *Deltaproteobacteria* | *uncultured* |
|  |  |  | 8 | *Flavobacteriia* | *Flavobacteriales* |
|  |  |  | 1 | *Gammaproteobacteria* | *Oceanospirillales* |
|  |  |  | 1 | *Gammaproteobacteria* | *uncultured* |
|  |  |  | 2 | *Planctomycetes Uncultured* |  |
|  |  |  | 2 | *Phycisphaerae* | *Phycisphaerales* |
|  |  |  | 2 | *Planctomicetacia-Pla3* |  |
|  |  |  | 3 | *Planctomycetacia* | *Planctomycetales* |
|  |  |  | 4 | *Verrucomicrobia-uncultured* |  |
| Yendegaia 2018 | 6508 | 250 | 6 | *Acidimicrobiia* | *Acidimicrobiales* |
|  |  |  | 2 | *Acidobacteria* | *Subgroup6* |
|  |  |  | 2 | *Alphaproteobacteria* | *Rhizobiales* |
|  |  |  | 11 | *Alphaproteobacteria* | *Rhodobacterales* |
|  |  |  | 14 | *Alphaproteobacteria* | *Rhodospirillales* |
|  |  |  | 4 | *Alphaproteobacteria* | *Rickettsiales-SAR116* |
|  |  |  | 4 | *Alphaproteobacteria* | *SAR11* |
|  |  |  | 1 | *Betaproteobacteria* | *Nitrosomonadales* |
|  |  |  | 1 | *Betaproteobacteria* | *Methylophilales* |
|  |  |  | 1 | *Cytophagia* | *Cytophagales* |
|  |  |  | 18 | *Deferribacteres* | *Deferribacterales-406* |
|  |  |  | 15 | *Deltaproteobacteria* | *Desulfobacterales* |
|  |  |  | 15 | *Deltaproteobacteria* | *SAR324* |
|  |  |  | 3 | *Flavobacteriia* | *Flavobacteriales* |
|  |  |  | 62 | *Gammaproteobacteria* | *Alteromonadales* |
|  |  |  | 17 | *Gammaproteobacteria* | *Oceanospirillales* |
|  |  |  | 1 | *Gammaproteobacteria* | *Pseudomonadales* |
|  |  |  | 1 | *Gammaproteobacteria* | *uncultured* |
|  |  |  | 1 | *Gammaproteobacteria* | *Vibrionales* |
|  |  |  | 1 | *Gammaproteobacteria* | *Xanthomonadales* |
|  |  |  | 18 | *Gemmatimonadetes* | *BD2-11* |
|  |  |  | 2 | *Chloroflexi-uncultured* |  |
|  |  |  | 5 | *Chloroflexi-SAR202* |  |
|  |  |  | 21 | *Thaumarchaeota-MGI* |  |
|  |  |  | 2 | *Cyanobacteria-uncultured* |  |
|  |  |  | 6 | *Phycisphaerae* | *Phycisphaerales* |
|  |  |  | 1 | *Sphingobacteriia* | *Sphingobacteriales* |
|  |  |  | 1 | *Thermoleophilia* | *Gaiellales* |
|  |  |  | 12 | *Thermoplasmata* | *Thermoplasmatales-MGII* |
|  |  |  | 1 | *Proteobacteria-uncultured* |  |
|  |  |  | 1 | *Verrucomicrobia uncultured* |  |
